# Effects of dietary supplementation with a mixed blueberry and grape extract on working memory in aged beagle dogs

**DOI:** 10.1017/jns.2017.33

**Published:** 2017-07-12

**Authors:** V. Fragua, A. Lepoudère, V. Leray, C. Baron, J. A. Araujo, P. Nguyen, N. W. Milgram

**Affiliations:** 1 Diana Pet Food, Elven, France; 2 Oniris, National College of Veterinary Medicine, Food Science and Engineering, Nantes, France; 3 CanCog Technologies, Toronto, Canada; 4 InterVivo Solutions Inc., Toronto, Canada

**Keywords:** Polyphenols, Blueberries, Grape extract, Cognition, Oxidative status, Aged dogs, Cognitive decline, CDS, cognitive dysfunction syndrome, DNMP, delayed non-matching to position, PEGB, polyphenol-rich extract from grape and blueberry, PEGB1, PEGB at 240 parts per million, PEGB2, PEGB at 480 parts per million

## Abstract

Cellular oxidative damage is thought to be one of the key mechanisms underlying age-related cognitive impairment in dogs. Several nutritional interventions to limit cognitive decline are reported in the literature. To our knowledge, the association of grape and blueberry extracts has never been tested in aged dogs. Our objective was to evaluate the effect of a polyphenol-rich extract from grape and blueberry (PEGB) on oxidative status and cognitive performances in aged dogs. A total of thirty-five beagle dogs (aged 8·0–14·5 years) were fed a basal diet with PEGB at either 0 parts per million (ppm) (*n* 11; control), 240 ppm (*n* 12; PEGB1) or 480 ppm (*n* 12; PEGB2) for 75 d. To investigate the effects of PEGB supplementation on cognition and oxidative status, a delayed non-matching to position (DNMP) test and RT-PCR on genes involved in oxidative stress were evaluated. The dogs fed PEGB1 showed a higher superoxide dismutase mRNA expression compared with dogs fed PEGB2 (*P* = 0·042) and with the control group (*P* = 0·014). Moreover, the dogs fed PEGB2 showed higher nuclear factor-like 2 (Nrf2) mRNA expression compared with the dogs fed PEGB1 (*P* = 0·027). Concerning the DNMP test, the proportion of dogs showing cognitive improvements relative to their baseline level was significantly higher in dogs fed the PEGB, regardless of the dosage, than in dogs receiving no supplementation (*P* = 0·030). The results obtained in the DNMP test suggested a potential benefit of the PEGB on working memory. However, this hypothesis should be further investigated to confirm this cognitive effect.

One of the most widely accepted theories of ageing is that the ageing process reflects oxidative stress characterised by a progressive accumulation of oxidative products and decreased endogenous antioxidant defence mechanisms. As a consequence, ageing is associated with changes in body condition and composition, energy requirements and metabolic activity, as well as the impairment of organ functions and immune status^(^^1^^)^. At the neuronal level age-related impairments affect morphological, chemical and functional properties, which decrease the efficiency of interneuronal connections. In pet animals, this can lead to impaired cognitive performances across multiple cognitive domains, such as memory, learning ability, attention, spatial abilities, as well as other processes^(^^2^^)^. As a consequence, senior pets undergo various behavioural changes perceived as problematic by their owners. The behavioural and cognitive changes reported when pets get older have been referred to as cognitive dysfunction syndrome (CDS)^(^^3^^)^. CDS has also been found to parallel many of the changes observed in human dementia and has been proposed to serve as a natural model of Alzheimer's disease^(^^2^^)^.

Nutritional interventions targeting cognitive decline in pets mainly focus on the prevention of age-related morphological and metabolic changes in the brain. To be efficient, this nutritional intervention must be started as early as possible and should also be used in combination with environmental enrichment^(^^4^^)^. Supplementation with antioxidant combinations^(^^4^^)^, phospholipids such as phosphatidylserine^(^^5^^)^, α-lipoic acid^(^^6^^)^, *S*-adenosyl-l-methionine^(^^7^^)^ or curcumin^(^^8^^)^ has been claimed to provide protection against oxidative and inflammation-induced damage in both brain and vascular tissues in aged dogs. On the other hand, another well-studied strategy in dogs consists of providing the brain with an alternative energy source in the form of medium-chain TAG^(^^9^^)^.

The present study focused on a nutritional strategy, namely, the use of polyphenols, which are phytochemical compounds well known for their antioxidant functions. In addition, polyphenols also interact with cellular pathways involved in chronic diseases such as neurodegenerative disorders^(^^10^^)^. Moreover, polyphenols are now highly studied for their potential beneficial effects on memory^(^^11^^)^.

To our knowledge, the association of blueberry and grape extract polyphenols has never been tested in aged dogs. For that purpose, the objective of the present study was to evaluate the effect of a new source of polyphenols, a polyphenol-rich extract from grape and blueberry (PEGB), on both (1) oxidative status via gene expression by RT-PCR in peripheral blood mononucleate cells (PBMC) and (2) working memory performance in aged dogs using a delayed non-matching to position (DNMP) test.

## Materials and methods

### Dogs and housing

A total of thirty-five beagle dogs (fifteen castrated males and twenty castrated females) aged from 8·0 to 14·5 years at study initiation were used in the present study.

All the dogs were in good general health based on an entrance health examination, cell blood count and clinical biochemistry work-ups. The dogs were group housed in 1·07 × 1·22 m^2^ pens with a maximum of four animals per pen in compliance with the Animal for Research Act, CanCog Technologies’ Local Institutional Animal Care and Use Committee, and the guidelines of the Ontario Ministry of Agriculture, Food and Rural Affairs. Environmental management, including temperature regulation, ventilation, humidity regulation and lighting, was provided, maintained and controlled in compliance with the same standards. Heating and cooling were electronically controlled and were set to maintain the animal room at a temperature range of 15–28°C. Fresh water was provided *ad libitum*.

### Experimental period and diets

The study was a longitudinal matched parallel-group blinded preclinical study. During baseline acclimatisation (days −27 to −1), all the dogs were fed using a control diet and were also assessed on the DNMP test (days −27 to −16), which was used to stratify the dogs to three cognitively equivalent treatment groups. From days 0 to 75, the dogs were fed a basal diet (PLB International; 3935 kcal/kg (16464 kJ/kg) calculated^(^^12^^)^ containing 26 % protein, 44 % fat and 30 % carbohydrates on an energy basis; Table 1), with the quantity fixed for each animal at a level that maintained their body weight^(^^12^^)^. For two groups, PEGB was included in the kibble at either 240 parts per million (PEGB1; *n* 12; 10 (sem 0·4) years; six males and six females) or 480 parts per million (PEGB2; *n* 12; 10 (sem 0·4) years; five males and seven females). There were also eleven animals in the control group (10 (sem 0·6) years; four males and seven females). Food intake was controlled daily.

**Table 1.** Ingredient, polyphenol and nutrient composition of the basal diet (Mean values with their standard errors for nutrient composition; *n* 3)

	Composition (%)	
	Mean	sem
Ingredient composition		
Yellow maize	21·8	
Wheat	21·7	
Maize gluten	20·0	
Poultry meal	14·3	
Poultry fat	11·4	
Beet pulp	6·5	
Dog digest liquid	2·0	
Fish meal	1·2	
Preservatives	0·5	
Sodium chloride	0·4	
Premix	0·2	
Polyphenol composition (ng/g)		
Anthocyanins	ND	
Catechins*	16·5	
Epicatechins*	ND	
Resveratrol	ND	
Total polyphenols (μg/g)	142·7	
Nutrient composition		
DM	92·0	0·19
Ash	4·9	0·15
Crude protein	29·3	0·46
Ether extract	20·2	0·97
Crude fibre	3·5	0·31

ND, not determined.

* Polyphenols corresponding to flavanol monomers.

The PEGB was a powder made of grape (*Vitis vinifera*) and blueberry (*Vaccinium angustifolium*) extracts containing specific polyphenols (27 % dry extract of total polyphenols; see Supplementary material S1).

### Delayed non-matching to position testing

DNMP testing was conducted on days −27 to −16 to determine the dogs’ baseline memory performance (12 d) and from days 58 to 63 (6 d) to evaluate the effect of the PEGB supplementation. Each trial was comprised of two phases: (1) a sample phase, where the dogs were required to displace an object placed over one out of three possible food well positions on a tray (the block to be displaced covered a food reward), and (2) a test phase, where dogs were presented with two identical objects after a delay (20 or 90 s); the one object was placed in the same position used in the sample phase and an identical object was placed in one of the two remaining positions (non-matching positions). In this phase, each dog was required to displace the object in the new position to get the food reward.

There were twelve trials per DNMP test session and there was one test session per d for all the dogs. For each test session, delays of 20 and 90 s were equally divided among the twelve trials. An inter-trial interval of 30 s was used. The dogs were tested on each of the designated days regardless of their score. During all the testing procedures, the animals were rewarded with Purina Essential Care Adult Formula wet canned dog food.

### Real-time PCR analysis

On days 0 and 75, approximately 8 ml of whole blood were collected by jugular venepuncture. PBMC were recovered by differentiated sedimentation rates using Histopaque^®^-1077 according to the manufacturer's instructions (Sigma-Aldrich). A quantity of 500 µl of TRIzol^®^ reagent (Gibco BRL) was added to PBMC and then frozen at −80°C. mRNA expression of catalase, nuclear factor-like 2 (Nrf2), glutathione peroxidise (GPx), hypoxia-inducible factor 1α (HIF1α), vascular endothelial growth factor (VEGF), NF-κβ, superoxide dismutase (SOD) and NADPH dehydrogenase quinone 1 (NQO1) were semi-quantified by RT-PCR (Supplementary material S2).

### Statistical analysis

All the analyses were conducted using SAS version 9.3 (SAS Institute Inc.).

Concerning mRNA expression levels, a mixed-effect model analysis for each gene was performed to evaluate treatment effects. The model included dog as the random effect, the fixed categorical effects of the baseline and treatment. Tukey's all-pairwise-comparison test was used to identify differences between the treatments. All the effects were tested at a 5 % level.

The DNMP analysis focused on the change in performance compared with baseline. The score used was the mean over the six testing sessions and the data were analysed using a mixed-effect model. The model included dog as the random effect, the fixed categorical effects of baseline, day, treatment, delay as well as delay × treatment interaction. All effects were tested at the 5 % level. Additionally, a χ^2^ test was performed on the number of dogs that showed improvement over baseline. For this analysis, the change from the post-treatment sum of scores of the two delays to the sum of scores of the baseline was calculated. If this difference was higher than 0, the dog's score improved from baseline. If the change was equal to 0, there was no change from baseline. If the change was lower than 0, the dog's score declined.

## Results and discussion

The present study is part of a larger project with the main goal of studying the benefits of a mixed blueberry and grape extract in aged humans and dogs (Neurophenol Consortium). In this experiment, we aimed at evaluating the effects of this ingredient on memory using the DNMP test and gene expression related to oxidative stress in aged dogs. A previous study from the Neurophenols Consortium project showed that some specific metabolites from PEGB polyphenols such as flavonoids (flavanols, anthocyanins and flavonols) and stilbenes (resveratrol) were found in dog plasma after ingestion^(^^13^^)^. In the present study these specific metabolites have not been measured. Whilst this represents something of a limitation, we hypothesise that PEGB supplementation in dogs would result in modified gene expression and enhanced memory function.

### Effects of polyphenols from grape and blueberry on mRNA expression of genes of oxidative state in aged dogs

There were no significant effects on either food intake (*P* > 0·100) or body weight (*P* > 0·100) between treatments. The results of gene expression are shown in Table 2. Whereas no changes in the mRNA expression of GPx, NF-κβ, VEGF, catalase, HIF1α and NQO1 were found among treatments (*P* > 0·100), the dogs fed PEGB1 showed a higher SOD mRNA expression compared with the dogs fed PEGB2 (*P* = 0·042) and with the control group (*P* = 0·014). These results are in concordance with previous reports in human subjects and rodents that demonstrate the ability of flavonoids, and more specifically flavanols, to elevate the activity of antioxidant enzymes, such as SOD and catalase, as a part of a regulation mechanism of reactive oxygen species^(^^14^^)^.

**Table 2.** mRNA expression from three groups of aged dogs fed a basal diet with a polyphenol-rich extract from grape and blueberry (PEGB) included at 0 parts per million (ppm) (control; *n* 11), 240 ppm (PEGB1; *n* 12) or 480 ppm (PEGB2; *n* 12) for 75 d (Adjusted mean values with their standard errors)

	Control		PEGB1		PEGB2		
	Adjusted mean*	sem	Adjusted mean*	sem	Adjusted mean*	sem	Treatment P*
Catalase	1·39	0·245	1·52	0·224	1·67	0·224	0·702
Nrf2	1·70^a,b^	0·319	1·14^b^	0·271	2·23^a^	0·283	0·035
SOD	0·97^b^	0·226	1·91^a^	0·217	1·12^b^	0·226	0·010
GPx	1·46	0·316	1·28	0·317	0·97	0·304	0·529
NQO1	1·77	0·275	1·82	0·264	2·23	0·283	0·621
HIF1α	2·58	0·305	2·37	0·317	1·98	0·316	0·398
VEGF	1·38	0·416	1·71	0·415	2·19	0·375	0·274
NF-κβ	1·53	0·232	1·30	0·222	1·65	0·222	0·548

Nrf2, nuclear factor-like 2; SOD, superoxide dismutase; GPx, glutathione peroxidise; NQO1, NADPH dehydrogenase quinone 1; HIF1α, hypoxia-inducible factor 1α; VEGF, vascular endothelial growth factor.

^a,b^ Adjusted mean values within a row with unlike superscript letters were significantly different (*P* < 0·05).

* Adjusted means, sem and *P* values come from mixed models performed for each mRNA expression of genes with baseline and treatment as fixed effects and dogs as the random effect.

Moreover, the dogs fed PEGB2 showed higher Nrf2 mRNA expression compared with dogs fed PEGB1 (*P* = 0·027). One of the most important mechanisms of the protection of neuronal cells against oxidative stress is the activation of the Nrf2 pathway. The binding of this transcriptional regulator with reactive oxygen species is the first step of the key mechanism regulating many genes’ expression, which activates the expression of several enzymes with antioxidant and detoxification capacities^(^^15^^)^.

### Effects of polyphenols from grape and blueberry on working memory in aged dogs

The analysis of the baseline DNMP performance (twelve sessions) was compared with the DNMP performances of the treatment phase (six sessions). The results of this analysis revealed a significant time effect (*P* = 0·001) and a significant delay effect (*P* < 0·001). Baseline performance on sessions 7–12 was significantly better than performance during sessions 1–6 (*P* < 0·001), which was expected because of practice effects.

Cognitive performances during the treatment phase were compared with the baseline cognitive performances in sessions 1–6. Accordingly, this comparison revealed a statistically significant effect of delay (*P* < 0·001) and no other significant main effects or interactions. On the other hand, the group means in baseline sessions 1–6 were also compared with the group means in test phase sessions 1–6. There was no significant effect of the polyphenol supplementation (*P* > 0·100). Likewise, no differences were found among treatments when the effect of delay on change in responding during the test phase was evaluated (*P* > 0·100).

The effect of PEGB supplementation was also evaluated by examining the number of animals in each group that showed improvement over their baseline. The proportion of dogs showing cognitive improvement relative to their baseline level was significantly greater in dogs fed PEGB, regardless of the dosage, compared with dogs receiving no supplementation (eight out of twelve, eight out of twelve and three out of eleven for dogs fed PEGB1, PEGB2 and the control, respectively; χ^2^ = 10·7, df = 4, *P* = 0·030).

The results using this χ^2^ statistical model suggested that PEGB improved memory. A significantly higher proportion of subjects from both supplemented groups showed improvements under the treatment condition on the DNMP task.

Dogs demonstrate reversal learning and DNMP impairments with increasing age^(^^2^^)^. Using DNMP performance, old dogs can be separated into three groups (unimpaired, impaired and severely impaired), in concordance with the various stages of Alzheimer disease in humans^(^^16^^)^. Siwak *et al.*^(^^17^^)^ demonstrated that some age-related behavioural changes, consistent with CDS, perceived by pet owners – such as sleep–wake cycle alterations, increased stereotypy, and decreased social contact with humans – are linked with DNMP impairments. Collectively, this suggests that memory impairment is an early consequence of canine ageing that precedes CDS.

Numerous studies in both animals and human subjects have shown that several polyphenol families present in the current tested extracts ameliorate learning and memory impairments, in particular flavanols^(^^18^^)^, anthocyanins^(^^19^^)^ and resveratrol^(^^20^^)^. In addition, Bensalem *et al.*^(^^21^^)^ showed that PEGB can play an important role on memory early in life in mice models. However, to our knowledge, this is the first time that supplementation with PEGB has been shown to potentially ameliorate memory impairments in aged dogs.

In summary, the results obtained on the DNMP suggested a potential benefit of the PEGB on working memory. This effect could be probably explained by the induction of expression of several genes associated with lower susceptibilities to oxidative stress. However, while expression of several genes may be enhanced with PEGB, further confirmation of anti-oxidant activity is warranted. Further studies, ideally with a larger population, are thus needed to confirm the appropriate dosage and cognitive-enhancing effects.

## Supplementary material

For supplementary material accompanying this paper visit http://dx.doi.org/10.1017/jns.2017.33. click here to view supplementary material
