# Supplementary material for: Effects of dietary supplementation with a mixed blueberry and grape extract on working memory in aged beagle dogs
Source: J Nutr Sci. 2017 Jul 12;6:e35. doi: 10.1017/jns.2017.33 (PMC5672304; doi:10.1017/jns.2017.33)
Supplement: Supplementary file 1 [file S2048679017000337sup.zip › S2048679017000337sup002.doc]

**Supplementary material S2**

**Reverse transcription followed by polymerase chain reaction. Materials and methods**

RNA was extracted using TRIzol reagent according to the manufacturers’ instructions (Invitrogen, Life Technologies, France). Total RNA concentration was quantified by spectrometry at 260 nm (Jenway, Genova Nano, UK), and samples with inappropriate 260/230 and 260/280 ratios were excluded. Reverse transcription was carried out in a 20µl reaction volume containing 1µg total RNA, hexamer primers, dNTP and Superscript III (according to the manufacturer’s instructions; Life Technologies). Real-time PCR was conducted in an ICycler System (MyIQ BioRad, Hercules, CA, USA) in a 20µl mixture containing 10µl MesaGreen Master Mix (Eurogentec, Angers, france) 1µl of sense and antisense primer (0.5mM each) and 2µl cDNA.

GAPdH was used as the housekeeping gene. The stability of the expression of this gene has been already assessed by comparison with other housekeeping genes. The primers were designed with Primer3 input progam. The sequences were: catalase: 5'-TGAGCCCAGCCCTGACAAAATG-3' (sense) and 5'-CTCGAGCCCGGAAAGGACAGTT-3' (antisense); Nrf2: 5'-GAAGTCTTCAGCATGCTCCG-3' (sense) and 5'-ACAACCCACTAGCTCGGAAA-3' (antisense); SOD: 5’-TGGTGGTCCACGAGAAACGAGATG-3' (sense) and 5'-CAATGACACCACAAGCCAAACGACT-3' (antisense); GPx: 5'-GCAACCAGTTCGGGCATCAG-3' (sense) and 5'-CGTTCACCTCGCACTTCTCAAAA-3' (antisense); NQO1: 5’-GTAGCGGCTCCATGTACTCT-3’ (sense) and 5’-TCTCCAGGCGTTTCTTCCAT-3’ (antisense); HIF1-α: 5'-CATGCTTTGGACTCTGACCA-3' (sense) and 5'-ATCCAATGCACTGTGGTTGA-3' (antisense); VEGF: 5'-TGAGGAGTTCAACATCACCATGC-3' (sense) and 5'-TTACACGTCTGCGGATCTTGTAC-3' (antisense); NFκB: 5'-CATCTACGACAGCAAAGCCC-3' (sense) and 5'-AATCCCCAAATCCTCCCCAG-3' (antisense); GAPdH: 5'-ACAGTCAAGGCTGAGAACGG-3' (sense) and 5'-CCACAACATACTCAGCACCAGC-3' (antisense). The fluorescence cycle threshold (Ct) was determined to quantify the relative amount of gene expression. Results are expressed according to the 2^-ΔΔCt^ method. Results were calculated compared to intern calibrator set before supplementation for each group.

Total RNA was quantified using a Nanodrop®ND-1000 spectrophotometer (Thermo Fisher, France). The quality and integrity of RNA were assessed using the 2100 Bioanalyzer and RNA 6000 Nano LabChip kit series II (Agilent Technologies, Les Ulis, France).
