# Supplementary material for: Effects of dietary supplementation with a mixed blueberry and grape extract on working memory in aged beagle dogs
Source: J Nutr Sci. 2017 Jul 12;6:e35. doi: 10.1017/jns.2017.33 (PMC5672304; doi:10.1017/jns.2017.33)
Supplement: Supplementary file 1 [file S2048679017000337sup.zip › S2048679017000337sup001.doc]

**Supplementary material S1**

**Composition of polyphenol-rich extract from grape and blueberry (PEGB) extract**

Polyphenol composition is presented as mean ± standard error of the mean (SEM; *n* 3)

| *Composition (% dry extract)* |  |
| --- | --- |
| Total polyphenols | 27 ± 3.0 |
| Anthocyanins | 0.18 ± 0.002 |
| Catechins* | 5.1 ± 0.40 |
| Epicatechins* | 4.3 ± 0.30 |
| Resveratrol | 0.53 ± 0.010 |

*Polyphenols corresponding to flavanol monomers
